# Immobilization of the Proteolytic Fraction P1G10 from *Vasconcellea pubescens* in Alginate–Chitosan Complex and Enzyme Activity Release

**DOI:** 10.3390/molecules30183747

**Published:** 2025-09-15

**Authors:** Jonathan Cisternas-Jamet, Verónica Plaza, Carlos Salas, Claudia Bernal, Luis Castillo

**Affiliations:** 1 Laboratorio de Bioquímica y Biología Molecular, Departamento de Biología, Universidad de La Serena, Av. Raúl Bitrán 1305, La Serena 1700000, Chile; jcisternas@userena.cl (J.C.-J.); vplaza@userena.cl (V.P.); 2 Departamento de Ingeniería en Alimentos, Universidad de La Serena, Av. Raúl Bitrán 1305, La Serena 1700000, Chile; 3 Departamento de Ciencias Básicas, Facultad de Ciencias, Universidad Santo Tomás, Panamericana Norte 1068, La Serena 1700000, Chile; 4 Centro de Investigación y Modelación de Negocios CIMON, Universidad Santo Tomás, Panamericana Norte 1068, La Serena 1700000, Chile; 5 Instituto de Ciencias Biológicas, Universidad Federal de Minas Gerais, Av. Antonio Carlos 6627, Belo Horizonte 31270-901, MG, Brazil; cesbufmg@yahoo.com; 6 Departamento de Química, Universidad de La Serena, Av. Raúl Bitrán 1305, La Serena 1700000, Chile

**Keywords:** P1G10, antifungal, chitosan, alginate

## Abstract

The proteolytic fraction (P1G10) from *Vasconcellea pubescens* displays pharmacological activity in diverse therapeutic settings. It is responsible for antifungal activity against *Botrytis cinerea*, impairing its germination and the integrity of the plasma membrane. The application of P1G10 is limited by stability in aqueous environments, where proteases lose activity. In this study, we aim to stabilize the proteolytic fraction, by complexation, to preserve the enzymatic activity ensued by controlled release. The proportion of each polymer, and the established reaction sequence, is chitosan (CS) plus P1G10 and alginate (ALG) using ALG:CS mass ratio = 1.0. Scanning electron microscopy (SEM) of the product shows the ALG-CS-P1G10 complex displaying a rough surface contrasting with the smoother surface of the ALG-CS complex, likely induced by interactions between the protein and ALG-CS complex. The optimal amount of protein taken up by the complex under this condition was 13 mg, and the incorporation yield was 72%. The melting temperature (Tm) determined by differential scanning calorimetry (DSC) in ALG-CS increased from 80 °C to 86 °C for the biocatalyst ALG-CS-P1G10; this difference was probably induced by the interactions between P1G10 and ALG-CS. Fourier transform infrared spectrometry (FTIR) comparison between ALG-CS and ALG-CS-P1G10 shows two bands in the biocatalyst at 1601 and 1523 cm^−1^, suggesting the presence of amine residues from P1G10 which is rich in lysine residues. The release of P1G10 from the complex was assessed by increasing the ionic strength in the media between 0.1 and 0.4 M NaCl. The results show that, at 0.3 M NaCl, the protein released after 8 h attained 70% and expressed enzymatic activity of 0.90 × 10^−3^ U/mg protein compared to the enzymatic activity from free P1G10 protein, which was 5.55 × 10^−4^ U/mg protein.

## 1. Introduction

The phytopathogenic fungus *Botrytis cinerea* causes gray mold disease on a wide range of plant species and is responsible for economic losses around the world [1,2,3]. The chemical control of *B. cinerea* is classically attained with synthetic antifungals containing different groups of fungicides such as dicarboximides, carboxamides or hydroxyanilides. However, the use of synthetic antifungals has been associated with hazardous effects for human health [4,5]. Therefore, current legislation limits the use of agrochemicals in favor of environmentally safer natural secondary metabolites which can be recycled. In recent years, plant metabolites p-hydroxybenzoic, caffeic, p-coumaric and ferulic acids displaying fungicide activity against [6] *B. cinerea* were described [7]. Also, essential oils from species such as clove, mustard [8], oregano, lavender and rosemary [9] display antifungal activity against *B. cinerea*.

Likewise, latex components of several plant species act as a defense against herbivores, fungi, parasites and even viruses [10]. The proteolytic fraction (P1G10) from *Vasconcellea pubescens* (ex *Carica candamarcencesis*) [11] contains 14 isoforms of cysteine proteinases (C1A papain family); the isoforms were characterized by their N-terminal sequence and amidase activity and do not contain known toxic components for human health [12,13,14]. P1G10 displays antifungal activity against *B. cinerea* by impairing its germination and the integrity of the plasma membrane [15]. Previous assays showed that exposure of P1G10 at 37 °C reduces its activity, affecting its pharmacological action [16]. The encapsulation of bioactive substances for industrial applications is an option in stabilizing the activity of bioactive species [17,18,19]. Many polymers can be used to encapsulate bioactive compounds for preventing food spoiling post-harvest [20]. Among them, chitosan and alginate stand out due to their intrinsic characteristics. Chitosan (CS) is a positively charged non-toxic, biocompatible and biodegradable polymer [21]. It is widely used in formulations, allowing the encapsulation and release of fertilizers and phytopathogenic agents, such as antifungals [22]. In parallel, sodium alginate (ALG), due to characteristics like CS, in addition to its gelling capacity, is a negatively charged polymer widely used in the encapsulation, preservation and release of bioactive compounds [23]. Recently, authors have studied the synergetic effects of CS and ALG on enzyme encapsulation [24] and vegetal extract [25], particularly on their stability and controlled release.

Recent studies further to expand the applicability and sophistication of alginate–chitosan protein delivery systems, ref. [26] demonstrating triggered protein release from calcium alginate–chitosan capsules in intestinal-mimetic conditions and confirming the modular release behavior of such matrices. Comparable systems have been used in advanced microscale platforms; the authors of [27] incorporated alginate–chitosan composites into dECM-based bioinks for cartilage-on-a-chip applications, while those of [28] designed a fully natural, pH-sensitive hydrogel system enabling targeted release in colon-simulated environments. A promising oral delivery approach was also reported by [29], who developed stable alginate–chitosan nanospheres for BSA encapsulation and controlled release under GI pH conditions. Moreover, ref. [30] demonstrated enhanced dermal delivery of protein hydrolysates using alginate–chitosan nanoparticles, supporting the versatility and translational potential of these biopolymeric systems.

In addition to its biotechnological relevance, P1G10 has been reported to exert antifungal effects against *B. cinerea*, the causative agent of gray rot in crops [15]. Since the direct application of P1G10 in a soluble form is limited, as the fraction loses activity when exposed to environmental conditions, the present study aims to encapsulate and stabilize P1G10 within an alginate–chitosan matrix, enabling controlled release of its proteolytic activity. This strategy not only provides a potential biofungicide of natural origin but also offers an environmentally friendly alternative to chemical antifungals, as the system is based on biodegradable and biocompatible polymers.

This study aims to optimize the encapsulation and stabilization of the proteolytic fraction P1G10 within alginate–chitosan matrices and to evaluate the subsequent release of its enzymatic activity. Specifically, we investigated the effect of polymer addition order, the optimal ALG/CS ratio, and the protein loading capacity of the system. By integrating encapsulation yield, structural/thermal characterization, and release kinetics, this work provides insights into the stabilization of P1G10 and its potential application as a bioactive biocatalyst in agri-food contexts.

## 2. Results and Discusion

### 2.1. Encapsulation of P1G10 with ALG/CS Complex

**Figure 1:**
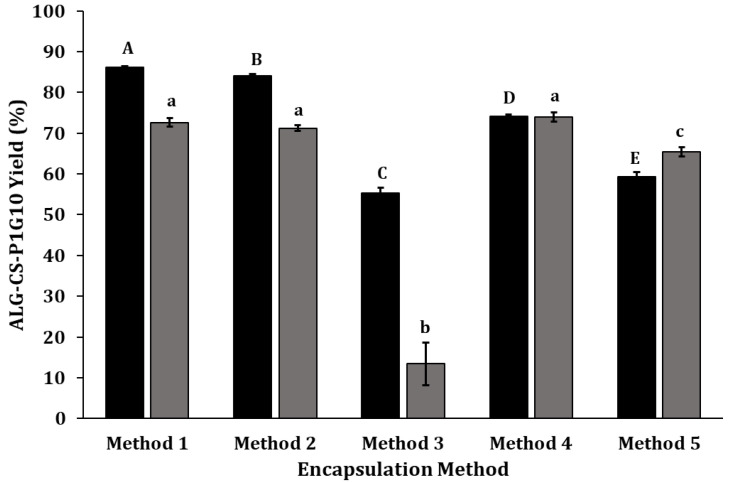
Efficacy of biocatalyst formation. The order in which each component is added represents methods 1–5, assessing the changes in the yield of biocatalyst. See Table 1 for the details of the addition order. 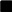, ALG-CS-P1G10 biocatalyst formation yield; 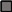, P1G10 protein encapsulation yield. Method 1 (CS → ALG → P1G10), method 2 (CS → P1G10 → ALG), method 3 (CS → P1G10 → ALG → CaCl_2_), method 4 (CS → P1G10 → ALG → CaCl_2_ → TPP) and method 5 (CS → P1G10 → TPP → ALG → CaCl_2_). Capital letters indicate statistically significant differences in the formation yield of the ALG-CS-P1G10 biocatalyst (*p* < 0.05), according to Fisher’s LSD test. Lowercase letters indicate statistically significant differences in the encapsulation yield of the P1G10 protein (*p* < 0.05), also according to Fisher’s LSD test.

CS and ALG complexes, plus tripolyphosphate (TPP) as a crosslinker at different conditions, are frequently employed to create CS beads [31], while calcium chloride (CaCl_2_) is a common crosslinker for ALG [32]. We searched for the conditions optimizing the encapsulation of P1G10. These results are summarized in Figure 1. The highest yield was observed in the absence of crosslinkers, using method 1 (CS → ALG → P1G10) and method 2 (CS → P1G10 → ALG) corresponding to 86.15% and 84.05%, respectively (Figure 1). A possible interaction between ALG-CS with P1G10 takes place, favoring encapsulation without the need of a crosslinker, similar to reports with other ligands [33]. The inclusion of crosslinkers affects the encapsulation yield (compare method 3, (CS → P1G10 → ALG → CaCl_2_), 4 (CS → P1G10 → ALG → CaCl2 → TPP) and 5 (CS → P1G10 → TPP → ALG → CaCl_2_) (Figure 1)). In method 3, the CaCl_2_ crosslinker decreases the encapsulation efficacy to 13.4%, influenced by the dominant complexation between CaCl_2_ and ALG, as reported by [34], preventing complexation between ALG and CS. On the other hand, the addition of TPP in method 4 (72.61%) attained levels like those in method 1 and 2, if P1G10 was included. When all components are added (CS → P1G10 → TPP → ALG → CaCl_2_), in method 5, as shown in Figure 1, the encapsulation yield was partly restored (>60%). In this case, the addition of TPP after P1G10 favors the interaction between P1G10 and CS.

**Table 1:** Order of addition of encapsulating components.

	Component				
Method	CS	P1G10	ALG	CaCl_2_	TPP
Amount (mg)	25.0	7.5	25.0	25.0	12.0
1	First	Third	Second	-	-
2	First	Second	Third	-	-
3	First	Second	Third	Fourth	-
4	First	Second	Third	-	Fourth
5	First	Second	Fourth	Fifth	Third

**Figure 2:**
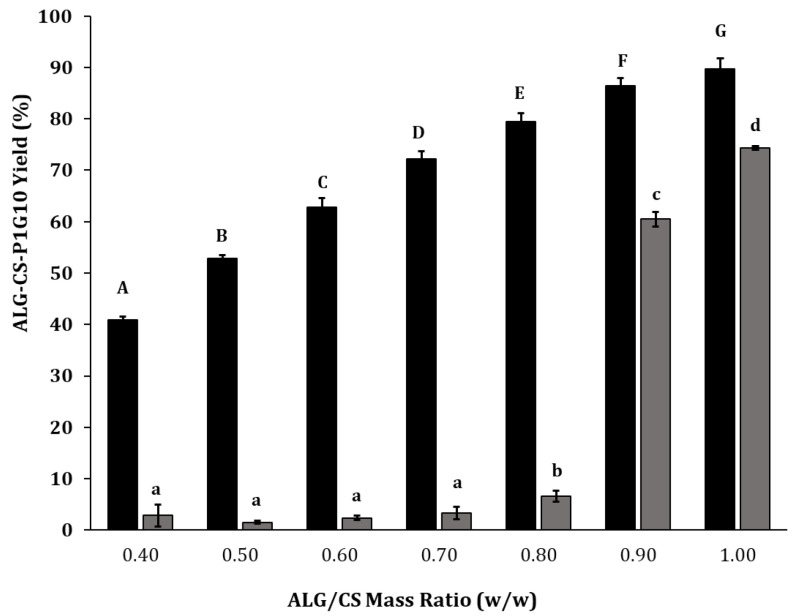
The effect of ALG/CS mass ratio changes on ALG-CS-P1G10 biocatalyst formation. 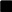, ALG-CS-P1G10 biocatalyst formation yield; 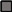, P1G10 protein encapsulation yield. Values are expressed as mean ± standard deviation. Different uppercase and lowercase letters indicate statistically significant differences (*p* < 0.05) in biocatalyst formation and protein encapsulation yields, respectively, as determined by Fisher’s LSD test.

Methods 3 and 4 were described by [35], using ALG-CS, with crosslinkers CaCl2 or TPP, as in this study. The authors suggest that using CaCl_2_ instead of TPP and low Mw CS at low mass ratios (˂2.4:1) improves the particle size and homogeneity of ALG-CS. According to [23], complexes containing CS and ALG with TPP or CaCl_2_ crosslinkers are stable and minimize ligand loss. In summary, these results show that methods 1, 2 and 4, as shown in Figure 1, afford the best protein encapsulation yield (≥70%). In preliminary trials, chitosan was also combined with other polyanions such as pectin, carboxymethylcellulose, and xanthan gum; however, these systems resulted in considerably lower yields or even negligible immobilization yields compared with ALG-CS, which achieved 74% (Supplementary Figure S1). Although method 1 exhibited improved yield, method 2 was selected, as fewer reactants were required, and it allowed the initial interaction of P1G10 and CS before complexation with ALG. The evaluation of various ALG/CS mass ratios (Figure 2) shows that, as the ALG/CS ratio rises, there is a gradual increase in the complex produced, from 40.86% at 0.40 to 89.68% at ratio = 1.0. In these experiments, ALG/CS at ratios >1.0 became partially soluble, preventing uniform complex formation. In contrast, the encapsulating yield of the biocatalyst containing P1G10 exhibits two patterns. The yield is >60% if the ALG/CS ratio is ≥0.9; meanwhile, this drops to 3.5% if the ALG/CS ratio is ˂0.7. Based on this data, we selected ALG/CS at ratio = 1 in subsequent experiments. Likewise, ref. [23] demonstrated that augmenting the ALG/CS ratio enhances the encapsulation efficiency when complexing a protease from *Aspergillus oryzae*. Similarly, when analyzing the results from encapsulating egg yolk immunoglobulin, it became evident that a ALG/CS ratio close to 2.0 generated larger encapsulation yields. Moreover, ref. [36] observed that ALG/CS ratios near 2.5 generate a higher encapsulation yield at 12.1% BSA protein and pH 5.5.

**Figure 3:**
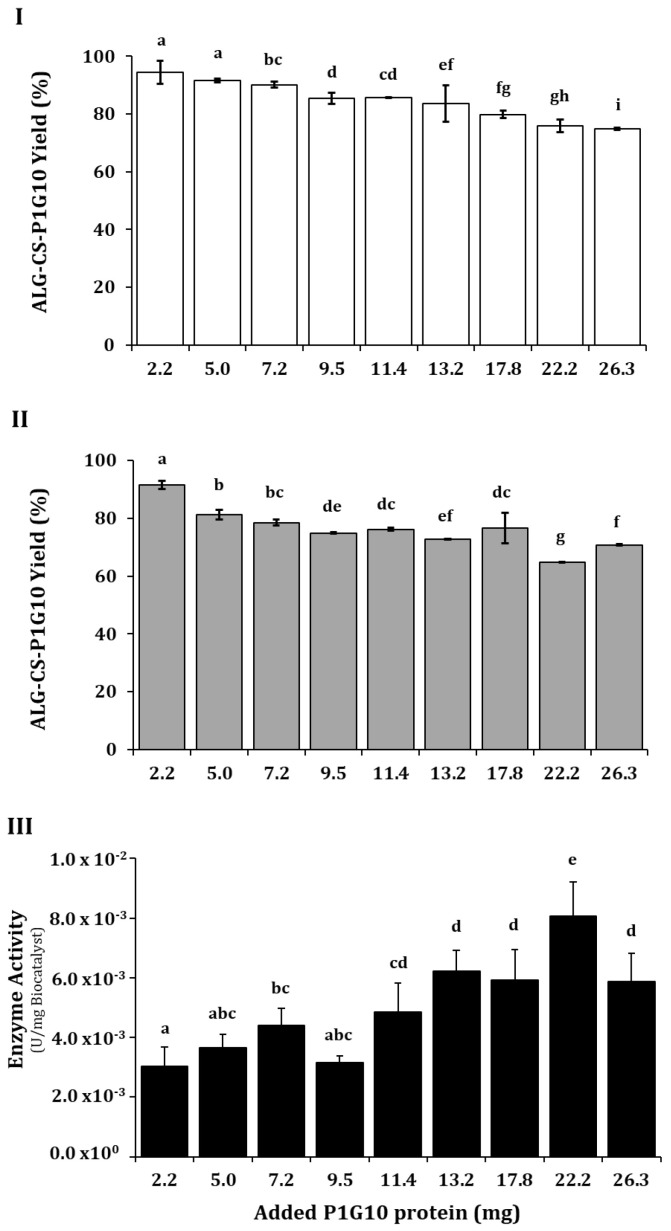
Effect of amount of P1G10 protein on the encapsulation efficacy. (**I**) (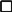), ALG-CS-P1G10 biocatalyst formation yield; (**II**) (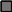), P1G10 protein encapsulation yield and (**III**) (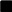), ALG-CS-P1G10 biocatalyst activity. Values are expressed as mean ± standard deviation. Different uppercase and lowercase letters indicate statistically significant differences (*p* < 0.05) determined by Fisher’s LSD test Based on the above results and the data from the literature [37], it is apparent that the amount of protein encapsulated influences biocatalyst formation and yield. In contrast, ref. [38] showed that by increasing the BSA protein from 1 to 8 mg/mL, the encapsulation yield decreased from 65 to 30% in ALG-CS microparticles. Similarly, their results show that the encapsulation yield decreases from 90 to about 15% when increasing the ALG/CS mass ratio from 1 to 5. Meanwhile, ref. [39] encapsulated BSA with ALG and CS, obtaining yields higher than 90% that remained high when the BSA/ALG mass ratio increased from 25 to 100. The disparity in yields is attributed to several factors influencing complexation between ALG-CS and the protein, including the molecular structure of the protein, its solubility, the ALG-CS protein mixing ratio, or the concentration and size of polyelectrolytes [40]. The authors of [41] pointed out that the choice of polyelectrolyte must include its charge density, molecular size, and pKa, while for the protein to be encapsulated, one should consider parameters such as pI, charge density, and hydrophobic distribution. The (pI) of P1G10 isoforms rank between 9.4 and 9.6 [42] and thus encompasses net positive charge over a wide pH range; CS has a pKa of 6.2 [22], suggesting a less favorable condition for electrostatic interaction at pH 5.1, as used in these experiments. On the other hand, the interaction between amino groups of CS and the carboxyl groups of ALG reported by [43] would allow trapping of P1G10, as shown by the encapsulation yields obtained at ALG/CS ratios 0.80–1.0. Based on the biocatalyst formation yield and the expressed enzyme activity, 13.2 mg of protein was selected in further encapsulation experiments.

Having established the reaction order and the appropriate ALG/CS ratio, we evaluated the amount of protein between 2 and 26 mg that affords the best incorporation into the complex and preserves enzyme activity. Figure 3 shows that increasing the protein moderately decreased the ALG-CS yield from 94 to 74% (Figure 3I) and ALG-CS-P1G10 yield from 90 to 70% (Figure 3II). Meanwhile, P1G10 between 2.2 and 9.5 mg did not have a significant effect on the enzyme activity (4.86 × 10^−3^ U/mg of complex) (Figure 3III); however, between 11.4 and 22.2 mg, it showed an increase in activity, attaining 8.05 × 10^−3^ U/mg at 22.2 mg. Clearly, the amount of bound protein does not reflect its activity when it becomes part of the complex and must, at least in part, represent the protein released by the complex.

### 2.2. Morphology and Physico-Chemical Attributes of AlG-CS-P1G10 Biocatalyst

**Figure 4:**
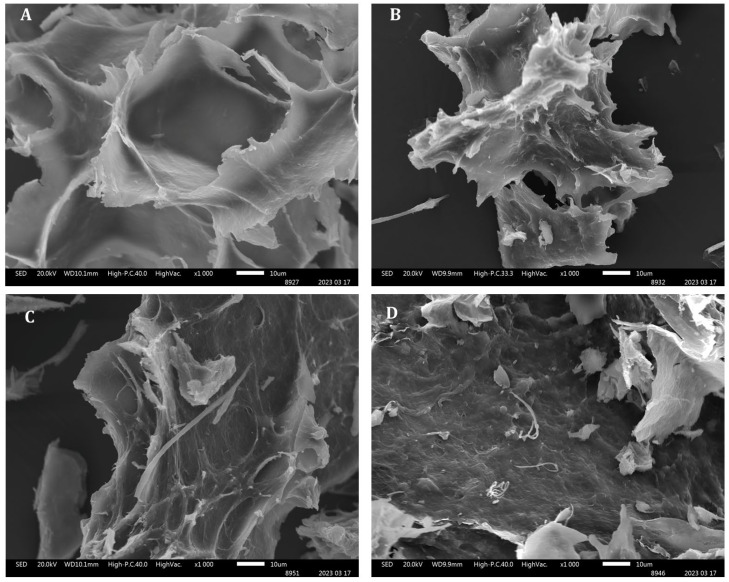
Scanning electron microscopy (SEM) analysis of samples. (**A**), ALG-CS complex ratio 0.8 *w*/*w*; (**B**), ALG-CS-P1G10 biocatalyst ratio 0.8 *w*/*w*; (**C**), ALG-CS complex ratio 1.0 *w*/*w*; and (**D**), ALG-CS-P1G10 biocatalyst ratio 1.0 *w*/*w*.

The morphology of the particles was analyzed by SEM images (Figure 4). Both ALG-CS and ALG-CS-P1G10 particles exhibit amorphous and irregular morphology. It is apparent that protein incorporation into ALG/CS mostly occurs at the surface of the particle; this can be seen by comparing Figure 4A (ALG/CS, ratio = 0.8) versus Figure 4B (ALG/CS + P1G10), and also Figure 4C (ALG/CS, ratio = 1.0) versus Figure 4B (ALG-CS-P1G10), where irregular spike projections can be seen at sites where protein is incorporated. These changes on the surface are attributed to interactions between P1G10 and the encapsulating media. These amorphous structures have been described when lutein is encapsulated with ALG-CS [44]. However, the ALG-CS-P1G10 biocatalyst at ratio 1.0 *w*/*w* (Figure 4D) shows increased irregularities on the surface, associated with enhanced interaction between P1G10 and the encapsulating media, which is consistent with the results in Figure 2, where the encapsulation yield of the protein increases from 3.3% at a ALG/CS ratio of 0.80 to 70% at a ALG/CS ratio of 1.0, representing an around 35-fold increase in the interaction between P1G10 and ALG/CS.

**Figure 5:**
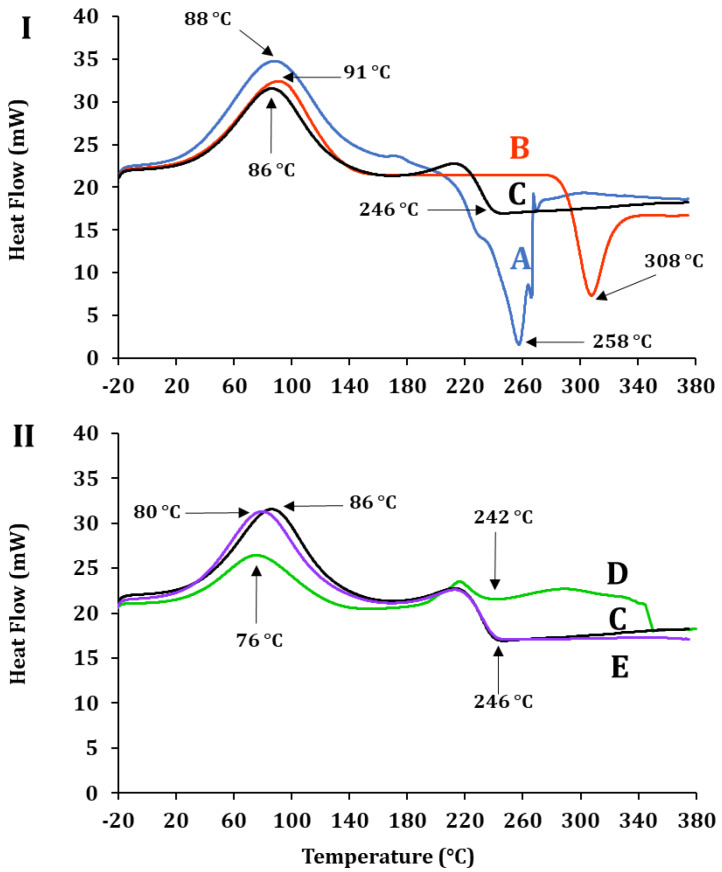
Differential scanning calorimetry of samples. (**I**): A, ALG; B, CS; C, ALG-CS complex. (**II**): C, ALG-CS complex; D, P1G10; E, ALG-CS-P1G10 biocatalyst.

Differential scanning calorimetry (DSC) evaluates the thermal properties of bioactive compounds inside matrices [45]. Figure 5I,II show the thermal profile of ALG-CS and its components and that of P1G10, before and after encapsulation. In Figure 5I, (A) displays the crystallization temperature of ALG, Tcr = 88 °C, CS (B), Tcr = 91 °C and ALG-CS (C) Tcr = 86 °C. The profiles and Tcr of each polymer are similar and confirm those reported by [43,46,47]. Instead, the melting temperature (Tm) of sample A is Tm = 258 °C, sample B, Tm = 308 °C, and complex AG-CS C = 246 °C. The differences suggest interactions between the AG and CS polymers affecting their physico-chemical properties. The authors of [43] reported a decrease in the Tm of the ALG-CS complex (83.6 °C) when compared to the mixture of both non-reacted polymers without reacting [48,49]. Figure 5II shows the thermograms of P1G10 protein (D), the biocatalyst E, and ALG/CS + P1G10, along with complex ALG/CS (C). Free P1G10 displays a crystallization temperature of 76 °C, while its Tm rose to 86 °C in the biocatalyst. On the other hand, encapsulated P1G10 and ALG/CS complex (E and C) share the same Tm 246 °C; however, the crystallization temperature of sample E (ALG-CS-P1G10) is 6 °C lower (80 °C) than ALG-CS (C), lacking the protein, which is 86 °C. It is suggested that the difference in Tms between C and E is caused by the interaction of the protein with the polymers during encapsulation and results from electrostatic interactions between the protein and polymers, as demonstrated by [43]. As shown in Table 2, the crystallization temperature (Tcr) of ALG-CS-P1G10 decreased by 6 °C compared to ALG-CS, suggesting the influence of P1G10 on the matrix structure. Although the melting temperature (Tm) of the biocatalyst remained unchanged (246 °C), this similarity may reflect the dominant thermal behavior of the polymer matrix. Nevertheless, the observed shift in Tcr, together with complementary evidence from FTIR, would support the hypothesis of specific interactions between P1G10 and the encapsulating polymers. To further confirm this interaction, FTIR analysis was subsequently carried out.

**Table 2:** Crystallization (Tcr) and melting (Tm) temperatures of ALG, CS, ALG-CS, P1G10, and ALG-CS-P1G10 samples determined by DSC.

Sample	Code	T_c_ (°C)	T_m_ (°C)
ALG	A	88	258
CS	B	91	308
ALG-CS	C	86	246
P1G10	D	76	242
ALG-CS-P1G10	E	80	246

**Figure 6:**
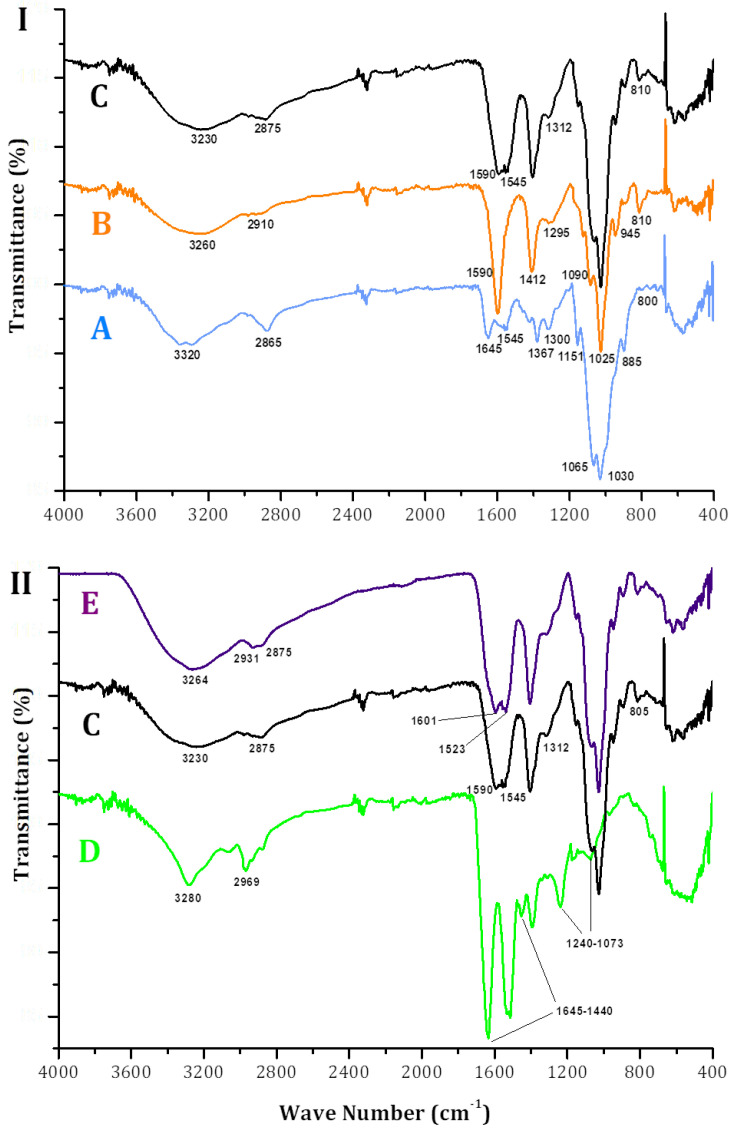
Fourier transform infrared spectra of samples. (**I**): A, ALG; B, CS; C, ALG-CS complex. (**II**): C, ALG-CS complex; D, P1G10; E, ALG-CS-P1G10 biocatalyst.

FTIR spectroscopic analysis confirmed crosslinking between CS, ALS and P1G10. Figure 6I shows the spectrum of CS (sample A) exhibiting bands at 1367, 1545 and 1645 cm^−1^ associated with primary, secondary and tertiary amides, respectively. On the other hand, the bands at 885, 1030, 1065 and 1151 cm^−1^ are associated with vibrations of C-O bonds, featured in polysaccharide structures such as chitin, which have been described by [50]. The broad band at 3320 cm^−1^ represents the overlap of hydroxyl and amine groups, while the band detected at 2865 cm^−1^ relates to methyl groups, as described by [50]. In the ALG spectrum (sample B), characteristic bands of polysaccharides are observed around 945, 1025 and 1090 cm^−1^, which are related to vibrations of C-O and C-O-C bonds. The bands detected at 1590 and 1412 cm^−1^ are associated with symmetric and asymmetric carboxyl groups, as previously described in the literature [51,52]. The bands detected at 2910 and 3260 are associated with the methyl and hydroxyl groups of the carboxyl groups, respectively. The spectrum of sample Figure 6, C, ALG-CS shows bands that are transferred to the complex coming from CS. For example, the band at 1545 cm^−1^ represents amino groups from CS emitted by the electrostatic interaction in ALG-CS. The band at 2875 cm^−1^ is more pronounced than in ALG Figure 6, B, probably due to the interactions of methyl groups of the CHS that are now part of ALG-CS in sample C.

In Figure 6II, the spectra of P1G10, the ALG-CS complex and the ALG-CS-P1G10 biocatalyst correspond to samples D, C and E, respectively. Due to the compound composition of the protein fraction, sample D is imprecise, which is attributed to specific chemical groups generating bands between 1600 and 1000 cm^−1^. However, we can suggest that the band at 1645 cm^−1^ represents amide and/or amine groups, since P1G10 isoforms encompass large amounts of positively charged lysine residues [53]. In addition, ref. [16] suggested that, in P1G10, bands between 1000 and 1241 cm^−1^ represent C-O-C interactions, and bands in the range between 1645 and 1440 cm^−1^, observed here too, represent aromatic residues (phe, tyr and trp). When comparing samples C and E in Figure 5II, it is evident in the latter that the two bands at 1601 and 1523 cm^−1^ are associated with the presence of amino acid residues in P1G10, as these bands are not evident in the spectrum of sample C. In addition, it is suggested that bands identified in Figure 5, E at 2.931 and 2.875 cm^−1^ are contributed by methyl residues from alkyl aminoacidic residues located in the protein, as these bands are not observed in ALG-CS sample (Figure 5, C). The additional bands at 1601 and 1523 cm^−1^ in Figure 5II, E, absent in the ALG-CS control (sample C), provide further evidence of the interaction between P1G10 and the encapsulating polymers. Together with the DSC data, these FTIR shifts support the hypothesis that electrostatic and structural changes occur upon encapsulation, influencing the thermal behavior of the system.

### 2.3. P1G10 Release: Kinetics, Enzyme Activity and Material Characterization

**Figure 7:**
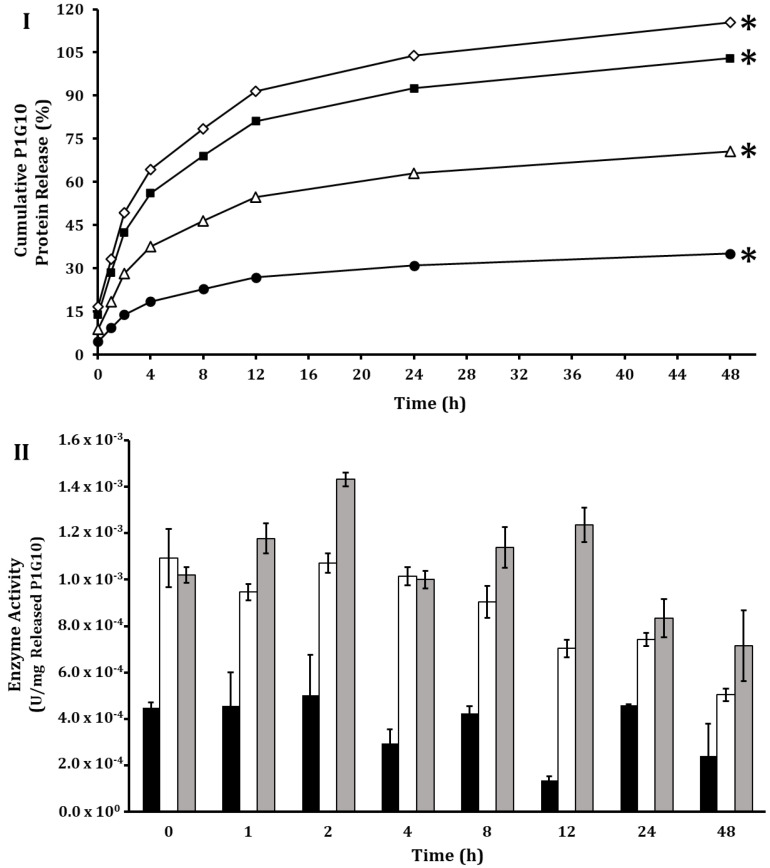
P1G10 protein release kinetics from ALG-CS-P1G10 biocatalyst as a function of sodium chloride concentration. (**I**), Cumulative release of P1G10 assessed by protein content at increasing NaCl concentrations: (●), 0.10 M; (△), 0.20 M; (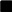), 0.30 M; (◇), 0.40 M. Release kinetics experiments were performed in triplicate. (*) indicates significant differences (*p* < 0.05) according to LSD test. (**II**), Enzymatic activity of released P1G10 at three NaCl concentrations: (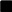), 0.20 M; (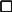), 0.30 M; (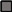), 0.40 M. No enzymatic activity was detected with NaCl 0.1 M.

The protein release from ALG-CS-P1G10 was analyzed between 0.1 and 0.4 M NaCl at various pHs during a 48 h interval (Figure 7). pH does not exert strong influence on protein release at pHs 3.5, 5.1 and 6.8. The authors of [33] evaluated the release of BSA from ALG-CS-BSA complex at pHs 1.2 and 7.4; they observed 20% release at 24 h but attained 78–100% within this interval at pH 7.4. The kinetics for release of P1G10 from the ALG-CS-P1G10 follows a hyperbolic function, attaining its maximum between 16 and 20 h, in which the amount of protein released increases as a function of NaCl, as described by [54]. No enzymatic activity of protein released was detected at NaCl 0.1 M.

The enhanced release of P1G10 from the ALG-CS-P1G10 complex at ionic strength ≥ 0.3 M NaCl supports the notion that encapsulation relies on ionic interactions between ALG-CS and P1G10. In subsequent assays, we adopted 0.3 M NaCl to induce protein release; at this concentration, 70% of the protein is freed to the medium in 8 h (Figure 7I). These results confirm that the release of P1G10 is primarily governed by electrostatic interactions; however, the protein is not merely adsorbed to alginate but entrapped within a polyelectrolyte complex formed by chitosan (positively charged amino groups) and alginate (negatively charged carboxyl groups). This cooperative network strengthens protein retention and explains why increasing ionic strength disrupts these interactions and promotes protein release [55,56]. To better understand the release process, the experimental data were fitted to classical mathematical models [57], including zero-order, first-order, Higuchi, Korsmeyer–Peppas, second-order, hyperbolic, and Weibull. Zero-order and first-order models showed poor fits, with R^2^ values below 0.9 and high SSE/RMSE values. In contrast, the Higuchi, Korsmeyer–Peppas, second-order, and Weibull models provided a much better description of the release kinetics (R^2^ > 0.9). Among them, the Weibull model consistently showed the highest R^2^ values and the lowest SSE and RMSE across all NaCl concentrations, indicating that it is the most suitable model to predict the release behavior of P1G10 from the ALG-CS matrix (Supplementary Table S1).

The enzymatic activity expressed per mg of released protein (Figure 7II) was equivalent to 0.904 mU/mg protein. The decline in the enzyme activity of freed protein requires that the ionic strength in the media be adjusted after release from the complex to prevent premature inactivation. It is important to note that, although NaCl (0.3 M) is needed to induce protein release, this requirement may limit the direct agronomic application of the biocatalyst, as high salt concentrations can negatively affect plant growth and seed vigor [58,59]. To overcome this limitation, alternative salts such as sodium citrate and potassium chloride are currently being evaluated as more compatible release agents.

**Figure 8:**
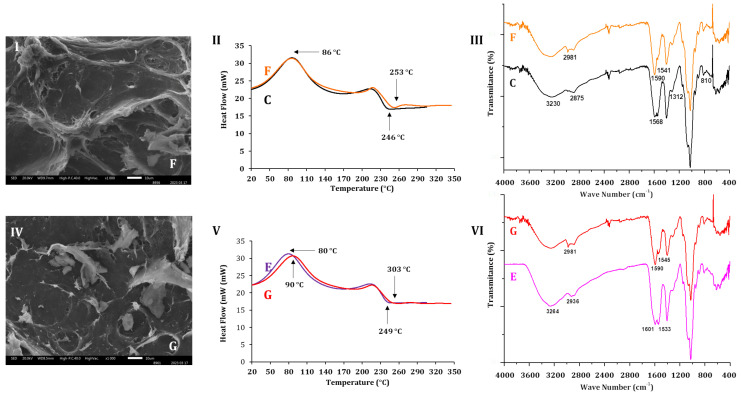
Effect of incubation on scanning electron microscopy (SCE) of ALG-CS and ALG-CS-P1G10 samples. Images represent the ALG-CS-P1G10 biocatalyst (**I**) before incubation and ALG-CS-P1G10 (**IV**) incubated with agitation for 24 h at room temperature in buffer acetate pH 5.1. The ALG-CS complex was prepared at a mass ratio of 1 *w*/*w*, as described in the Methods Section. Differential scanning calorimetry (DSC) of ALG-CS and ALG-CS-P1G10 samples. In panel (**II**), the ALG-CS complex (F) shows the sample after incubation and C the same sample before incubation. In panel (**V**), E represents the ALG-CS-P1G10 sample before incubation and G the same sample after incubation. Fourier transformed infrared resonance (FTIR) of ALG-CS and ALG-CS-P1G10 samples. In panel (**III**), (F) depicts the ALG-CS complex after 24 h incubation and C the same sample before incubation. In panel (**VI**), G shows the ALG-CS-P1G10 sample after 24 h incubation and E the sample before incubation.

Figure 8I,IV show the changes in morphology assessed by SEM of the ALG-CS complex (sample F) and ALG-CS-P1G10 (sample G) when incubated at RT with orbital agitation for 24 h in buffer acetate pH 5.1. By comparing Figure 4C with Figure 8I, the latter shows more irregularities, and the emergence of breaks, associated with shearing due to agitation. In contrast, the ALG-CS complex at time 0 (Figure 4C) depicts a smoother surface and an absence of or diminished gaps. The shearing effect caused by incubation in aqueous medium has been observed already [60], in BSA encapsulated into ALG-CS hydrogels incubated at 37 °C in PBS buffer without stirring, with pore formation seen during the incubation period.

The DSC thermograms in Figure 8II compare the ALG-CS complex before incubation (sample C) and after incubation at RT (sample F). Both samples exhibited the same crystallization temperature (Tcr = 86 °C), indicating that incubation period did not alter this parameter. However, the melting temperature (Tm) increased from 246 °C in sample C to 253 °C in sample F, suggesting structural rearrangements in the polymer matrix upon incubation. In Figure 8V, biocatalyst samples before (E) and after 24 h incubation (G) are compared. In this case, the crystallization temperature increased from 80 °C (sample E) to 90 °C (sample G), indicating a stabilizing effect on the matrix after exposure to shear stress. Furthermore, the Tm rose from 249 °C (sample E) to 303 °C (sample G), denoting a more pronounced shift than that observed in the ALG-CS complex (C → F), suggesting that incorporation of P1G10 into the ALG-CS matrix provides additional structural stabilization during incubation, likely mediated by protein–polymer interactions (Figure 5I). To further corroborate these thermal observations, FTIR analysis was performed to assess potential structural modifications and protein–polymer interactions.

Figure 8III shows the initial FTIR spectra of ALG-CS complex before incubation (C) and ALG-CS after incubation (F). In the spectrum of sample F, a band at 1541 cm^−1^ is observed and associated with CS, the spectrum of which has bands at 1545 cm^−1^ induced by secondary amines. Another band standing out in sample F, at 2981 cm^−1^, relates to the increase in methyl groups that, in spectrum B of Figure 6I, is observed at 2910 cm^−1^. On the other hand, ALG-CS-P1G10 after incubation ((sample G) also shown in the SEM image (Figure 8IV)) displays wear produced by the shear friction generated during incubation, if compared with the SCE image in Figure 4D. However, in this case, the effect is less pronounced than in ALG-CS sample after incubation (sample F), probably due to the presence of the protein incorporated as part of the biocatalyst. In the thermograms in Figure 8V, the Tcr of the biocatalyst following incubation was 90 °C, while the same sample before incubation (sample E) had lower Tcr (80 °C). For Tm, ALG-CS-P1G10 before incubation (sample E) attained a Tm of 249 °C, like in the thermogram of Figure 5II (246 °C); however, the Tm of sample G (CS-ALG-P1G10) attained 303 °C after the incubation interval. The spectrum of ALG-CS-P1G10 sample G in Figure 8VI depicts bands at 1545 cm^−1^ and 1590 cm^−1^ associated with CS (Figure 6, A); they depart from bands in ALG-CS-P1G10 sample E (VI) at 1601 and 1533 cm^−1^ that are associated with functional groups from the protein. As in sample F (III), sample G (VI) exhibits a band at 2981 cm^−1^ linked to methyl groups. The results imply that the biocatalyst undergoes attrition caused by agitation, which favors disruption of the encapsulating particle. Despite the ALG-CS-P1G10 (sample G) being subjected to agitation during the incubation, the protein did not leak or release (Supplementary Figure S2) into the aqueous medium. Therefore, it is probable that the protein, ALG and CS generate stable interactions, preserving the protein complex. For instance, the biocatalyst loaded with 11 mg of P1G10 released approximately 8 μg protein/mg biocatalyst after 48 h of incubation, which represents a negligible fraction (<0.1%) compared to the initial load. This release is likely attributable to the desorption of surface adsorbed protein, while the bulk of the protein remains retained within the polymeric matrix. Therefore, it is plausible that P1G10, ALG, and CS generate stable interactions that preserve the integrity of the biocatalyst. In particular, the band observed at 1541 cm^−1^ in sample F (Figure 8III) is attributed to secondary amine groups and corresponds to the 1545 cm^−1^ band already present in the ALG-CS spectrum in sample C (Figure 8II) and the CS control (sample A, Figure 6I), confirming its assignment to chitosan functional groups. Similarly, the band at 2981 cm^−1^ in sample F (Figure 8III) can be associated with methyl group vibrations. This band presents a shift when compared with the signals detected at 2910 cm^−1^ in ALG (sample B, Figure 6I) and 2875 cm^−1^ in ALG-CS (sample C), suggesting structural modifications in the polymer matrix upon incubation. These assignments are consistent with standard FTIR data available and with previous reports on polysaccharide–protein interactions [50,51,52].

**Figure 9:**
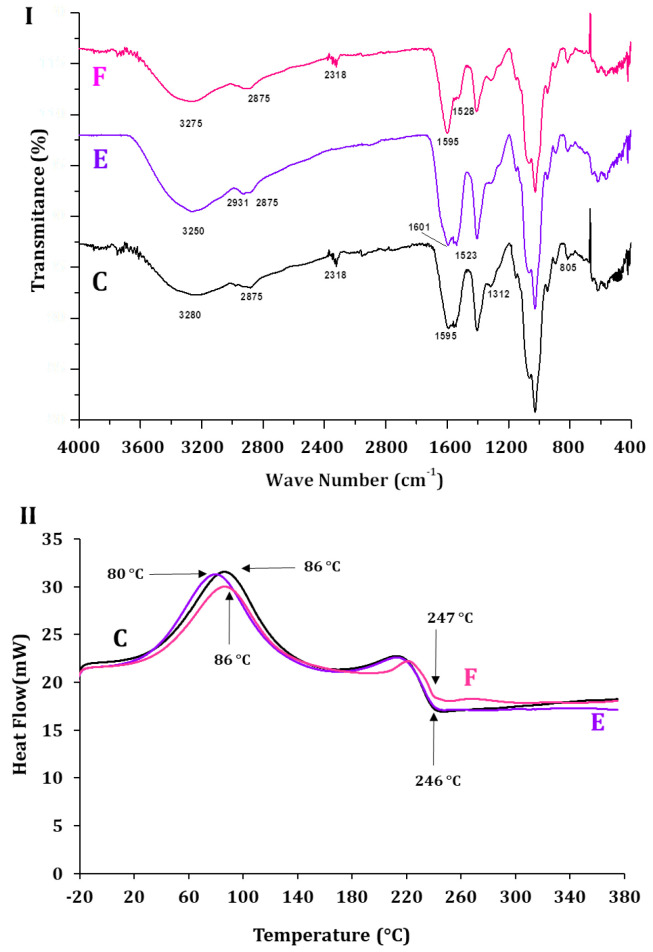
Evaluation of P1G10 release from ALG-CS-P1G10. (**I**) Fourier transform infrared (FTIR) and (**II**) differential scanning calorimetry (DSC) of samples: C, ALG-CS complex; E, ALG-CS-P1G10; F, ALG-CS-P1G10 after 48 h in acetate buffer with 0.3 M sodium chloride solution.

The spectrum in Figure 9I shows the ALG-CS-P1G10 biocatalyst, after 48 h incubation in buffer containing 0.30 M NaCl (sample F), lacking the differentiated bands at 1601 and 1523 cm^−1^ found in ALG-CS-P1G10 before incubation (sample E). It also lacks two bands seen in sample E at 2931 cm^−1^. It can be assumed that the spectrum of F approaches that of C (ALG-CS complex) but with modifications associated with the partial disruption of the polymers that initially formed the biocatalyst, since the band at 1595 cm^−1^ in C is more intense than in F. The spectrum of F was like ALG (Figure 6I (B)), suggesting that the relaxation of complex ALG-CS-P1G10 is an intrinsic interaction. Similarly, the thermograms of samples (C, E and F) in Figure 9, II show that the Tm of sample F is 86 °C, like in sample C. In other words, the Tm of the complex after protein release approaches that of the complex containing just ALG and CS. A similar situation is observed in samples C and F that share a Tm of 246 °C instead of 247 °C.

Finally, to explore the potential applicability of the ALG-CS-P1G10 biocatalyst, preliminary antifungal assays were performed against *B. cinerea*. As shown in Supplementary Figure S3, the biocatalyst inhibited the mycelial growth of the phytopathogen under laboratory conditions, whereas neither the control nor the ALG-CS matrix alone had a comparable effect. These findings suggest that the encapsulated formulation retains the bioactivity of P1G10 and can exert a protective effect against fungal proliferation, thereby reinforcing the potential of this system as a natural biofungicide; however, further studies will be required to demonstrate the effectiveness of ALG-CS-P1G10 biocatalyst in the field.

## 3. Materials and Methods

### 3.1. Papaya Latex Collection

Latex collection (*Vasconcellea pubescens*) was carried out between March and December 2021 at the farm “Delicias de Huentelauquén” in the Huentelauquén sector (−31.596, −71.545), 189 km from La Serena, Chile. Unripe papaya fruits were selected, and the dust was removed from the surface with paper towel wetted with 70% *v*/*v* ethanol solution prior to collection. Subsequently, 3–4 longitudinal incisions 3 mm in depth were made on the fruit surface with a steel scalpel between 08:00 and 09:00 am, as described by [12]. The latex material was stored at −80 °C before freeze-drying.

### 3.2. Protein Extraction from Freeze-Dried Papaya Latex

The protein extraction process, as previously described by [14], was performed with some modifications. First, 15.0 g of lyophilized papaya latex was mixed with 50 mL of the extraction buffer (0.010 M EDTA, 0.020 M Cysteine-HCl, 0.0020 M DTT and 0.60 M sodium acetate pH 5.1). The mixture was incubated with stirring for 30 min at room temperature and centrifuged for 10 min at 8.000 rpm. The supernatant was loaded onto a chromatographic column with Sephadex G-10. The filtration of P1G10 was performed according to the methodology described by [15]. The identity of the obtained P1G10 fraction was confirmed by size-exclusion chromatography and SDS-PAGE. As shown in Supplementary Figure S4A, the chromatogram obtained by purifying the latex extract on a Sephadex G-10 column is consistent with the profile described by [14]. The protein composition and stability of the fraction were established by protein electrophoresis and enzyme activity dosage, as described by [61], confirming that the preparation used in this study corresponds to the standardized proteolytic fraction P1G10 (Supplementary Figure S4B).

### 3.3. Encapsulation of the Proteolytic Fraction P1G10

The polymer solutions were prepared according to methodologies described by [62,63]. Chitosan (CS, CAS 9012-76-4) was obtained from Merck-Sigma Aldrich (St. Louis, MO, USA) and corresponds to a low-molecular-weight grade with a degree of deacetylation > 75% and a viscosity of 20–300 cps (1% *w*/*w* solution in 1% acetic acid). Sodium alginate (ALG, CAS 9005-38-3), purchased from Loba Chemie (Mumbai, India), is a food-grade sodium polymannuronate (≥91%) with a minimum viscosity of 30 cps (1% *w*/*w* solution in water). All other reagents were of analytical grade and used as received. A solution of low Mw CS (Merck-Sigma Aldrich) at 2.5 mg/mL in 10% *v*/*v* acetic acid was prepared. ALG solution (Loba Chemie, Mumbai, India) was prepared at 2.5 mg/mL in distilled water. Both solutions were dissolved with stirring at 150 rpm for 7 h and filtered through a 0.45 μm cellulose ester filter at room temperature (20 °C).

The encapsulation of P1G10 was performed as outlined by [23], mixing similar amounts (ALG/CS mass ratio = 1) of ALG and CS plus 13.2 mg of protein (P1G10), the latter equivalent to 52% of each component of encapsulating medium. The mixture was incubated at room temperature with stirring for 10 min. Then, 10 mL of 2.5 mg/mL ALG was added through an infusion pump, at a flow rate of 5.0 mL/min with stirring, followed by 10 min additional stirring. The mix was centrifuged at 8000× *g* for 30 min at 14 °C. An aliquot of the supernatant (5 mL) was withdrawn for analysis; the pellet was freed from unbound protein with a small volume of 0.1 M acetate buffer at pH = 5.1 and the remaining sediment was lyophilized to dryness.

### 3.4. Protein Quantification

Protein quantification was carried out according to [64] using, as standard, a solution of bovine serum albumin at 1 mg/mL. To establish the best amount of protein that can be encapsulated, increasing amounts of P1G10 were added into the mix, between 2 and 26 mg at a constant ALG/CS mass ratio = 1.0, as described by [65]. The loaded protein was measured on the final supernatant using the Lowry assay, as described above.

### 3.5. Determination of Enzyme Activity

Enzyme activity was determined according to the methodology outlined by [66]. In general, 50 μL of supernatant from the encapsulation mix (or P1G10) in a 1 cm thick cell was reacted with 20 μL of a solution of BOC-Ala-ONp substrate from Sigma Aldrich (St. Louis, MO, USA) in 2000 μL of 0.1 M phosphate buffer, pH = 6.8, at a temperature of 37 °C under constant stirring in a double-beam spectrophotometer equipped with stirring and temperature control. The absorbance was recorded at a wavelength of 348 nm for 200 s and the obtained slopes were used to determine the enzyme activity units over the mass of the biocatalyst used according to the following expression:

$$AEE=\left(\frac{U}{Biocat}\right)=\frac{\frac{A}{t}}{E\cdot l}\cdot V_{T}\cdot \frac{1}{V_{E}}\cdot \frac{1}{B_{m}}$$

where AEE represents the specific enzyme activity, U/Biocat corresponds to the units of enzyme activity per milligram of biocatalyst (μmol p-Nitrophenol/min∙mg Biocatalyst), A/t represents the absorbance over reaction time (slope obtained from the spectrophotometer, abs/min), ℰ corresponds to the molar extinction coefficient from BOC-Ala-ONp (5235∙^10−3^ L/μmol∙cm), ℓ represents the path of the spectrophotometric cuvette (1 cm), VT represents the total reaction volume (μL), VE represents the volume of enzyme solution (supernatant) used in the reaction (μL) and Bm corresponds to the mass of biocatalyst in mg.

### 3.6. Evaluation of Encapsulating Parameters

Although the processes of encapsulation by ionic gelation and complexation using polyelectrolytes are described in the literature [67], some authors alter the order for the addition of polymer, the crosslinking agents [35], and the enzyme to be encapsulated [68].

To assess the potential effects of tripolyphosphate, calcium chloride, and ALG/CS on the yield of ALG-CS-P1G10, several combinations of these components were evaluated (Table 1).

First, Second, Third, etc., reflect the order for the addition of each reagent. (-) indicates a lack of reagent. The volume of the reaction mix is 20 mL in 0.1 M acetate buffer at pH 5.1. Chitosan was selected as the first component of the immobilization sequence due to its higher viscosity and lower solubility at the concentration used, which complicates mixing if added later. Pre-mixing chitosan with P1G10 facilitates protein–polymer interactions and, upon subsequent addition of alginate, promotes the formation of a stable polyelectrolyte complex that improves protein entrapment.

### 3.7. Determination of the Mass Ratio ALG/CS

To establish the mass ratio of the encapsulant media allowing the appropriate formation of the biocatalyst (ALG-CS-P1G10), the methodology used by [69] was applied with some modifications. For this purpose, different ALG/CS mass ratios were evaluated by keeping the CS concentration constant at 2.5 mg/mL (maximum solubility in 10% *v*/*v* acetic acid) and varying the ALG concentration from 1.0 to 2.5 mg/mL (stable range as a colloidal solution), generating mass ratios between 0.40 and 1.0. The generated product was evaluated in terms of biocatalyst formation yield and the encapsulated protein yield.

The biocatalyst formation yield was determined according to the following expression:

$${Y\left(\%\right)}_{ALG/CS}=\frac{M_{B}}{M_{ALG}+M_{CS}{+M}_{P1G10}}\times 100$$

in which Y(%)ALG/CS represents the yield of biocatalyst formed, MB corresponds to the mass of biocatalyst formed, MALG is the mass of ALG, MCS is the mass of CS and MP1G10 is the mass of P1G10 protein.

The encapsulation yield was determined according to the following expression:

$${Y(\%)}_{ALG-CS-P1G10}=\frac{M_{0}-M_{S}}{M_{0}}\times 100$$

where Y(%)ALG-CS-P1G10 represents the encapsulation yield, M0, the mass of protein added and MS, c the mass of protein present in the supernatant (after encapsulation). The masses are expressed in milligrams (mg).

### 3.8. Scanning Electron Microscopy (SEM)

Imaging by scanning electron microscopy was performed according to [60] with some modifications. The samples were powdered on a dual-phase carbon adhesive mounted on a cylindrical bronze sample holder. They were then metallized with a 30 nm thick gold layer on a Fine Coat Ion Sputter JEOL JFC 1100 (Tokyo, Japan). Images were produced using secondary electrons at 15 Kv in a JEOL JSM IT300LV scanning electron microscope (Tokyo, Japan).

### 3.9. Differential Scanning Calorimetry (DSC)

Changes in the transition temperature during the formation of the complex by the interaction of ALG, CS and P1G10 were measured by calorimetric assay, as described by [44], using differential scanning calorimetry Perkin-Elmer DSC 4000 (Waltham, MA, USA). A total of 11 mg of each polyion and the formed biocatalyst were sampled in aluminum capsules. Sample heating between −20 and 350 °C was set at 5 °C/min, at a constant rate, under a N_2_ atmosphere and flow rate of 20 mL/min.

### 3.10. Fourier Transform Infrared Spectrometry (FTIR)

To evaluate the interactions between ALG, CS and P1G10, Fourier transform infrared spectrometry (FTIR) was performed, as described by [70], on a Spectrum Two Perkin-Elmer infrared spectrometer (Waltham, MA, USA) from 4000 to 400 cm^−1^ at 4 cm^−1^ resolution. The analyzed samples included ALG, CS, ALG/CS complex, P1G10 and the biocatalyst ALG-CS-P1G10.

### 3.11. Release Kinetics

The protein was released as described by [71] with some modifications. The biocatalyst (100 mg) was incubated with the release media (buffer acetate pH 5.1) at various concentrations of NaCl to assess the release of P1G10 in 20 mL vials. The release was initiated by addition of 10 mL 0.1–0.4 M NaCl in aqueous solution at room temperature and constant agitation. A 1.0 mL aliquot from each sample was withdrawn at 0, 1, 2, 4, 8, 12, 24 and 48 h intervals. An equal volume of NaCl (1 mL) of the same concentration was added back to the vial to keep the reaction volume constant. Each experiment was performed in triplicate. The amount of protein released was determined by Lowry assay [64] and calculated according to the following expression:

$$Q\left(\%\right)=\left[\frac{C_{n}\cdot V\cdot V_{i}\sum_{i=0}^{n-1}C_{i}}{B_{m}\cdot {Y(\%)}_{ALG-CS-P1G10}}\right]$$

in which Q (%) corresponds to the percentage of protein released, Cn is the concentration of the sample taken at time n, V is the total reaction volume (reactor system), Vi is the spot sample volume, Ci is the concentration of the spot sample, Bm corresponds to the mass of biocatalyst used and (%)YALG-CS-P1G10 represents the encapsulation yield. In parallel, the residual enzymatic activity of each aliquot was measured according to the protocol described above.

## 4. Conclusions

In this study, we demonstrated that the encapsulation of the proteolytic fraction P1G10 into an alginate–chitosan (ALG-CS) matrix provides both stabilization and the controlled release of the protein. The structural and thermal analyses (SEM, DSC, and FTIR) revealed that the observed morphological changes in the biocatalyst surface are attributable to electrostatic and steric interactions between P1G10 and the polymer network. These interactions account for the enhanced thermal stability and modified crystallization behavior detected by DSC, as well as the additional spectral bands detected by FTIR. From a functional perspective, the release assays confirmed that protein liberation is primarily governed by ionic strength, consistent with the ionic interactions underlying encapsulation. Importantly, while free P1G10 rapidly loses activity under similar conditions, encapsulation preserved enzymatic function and enabled a sustained release profile.

Overall, the encapsulation of P1G10 in an ALG-CS matrix not only improves its stability and activity but also provides a practical strategy to overcome the intrinsic instability of free proteases in aqueous environments. This work highlights the potential of biopolymer-based delivery systems to enhance the applicability of proteolytic enzymes in biotechnological and biomedical contexts, where controlled activity and extended stability are essential. In addition to serving as a protective storage matrix, the ALG-CS system can also function as a delivery platform for P1G10 in agri-food contexts. Preliminary antifungal assays suggest that the encapsulated protein is able to inhibit the growth of *B. cinerea*, reinforcing its potential use as a natural biofungicide. Thus, the immobilization approach described here combines both protein stabilization and functional applicability against phytopathogens.

## 5. Patents

Patent Application Number 202502137 (INAPI-Chile).

## Data Availability

The original contributions presented in the study are included in the article/Supplementary Materials; further inquiries can be directed to the corresponding authors.

## Supplementary Materials

The following supporting information can be downloaded at: https://www.mdpi.com/article/10.3390/molecules30183747/s1, Figure S1: Encapsulation performance of P1G10 protein; Figure S2: Effect of incubation on ALG-CS-P1G10 biocatalyst release; Figure S3: Effect of ALG-CS-P1G10 biocatalyst concentration on *Botrytis cinerea* growth; Figure S4: Purification and characterization of the proteolytic fraction P1G10; Table S1: Mathematical modeling of P1G10 release; Table S2: Statistical analysis of P1G10 protein release for each time and NaCl concentration.
