# Supplementary figures and images for: Immobilization of the Proteolytic Fraction P1G10 from Vasconcellea pubescens in Alginate–Chitosan Complex and Enzyme Activity Release
Source: Molecules. 2025 Sep 15;30(18):3747. doi: 10.3390/molecules30183747 (PMC12472595; doi:10.3390/molecules30183747)

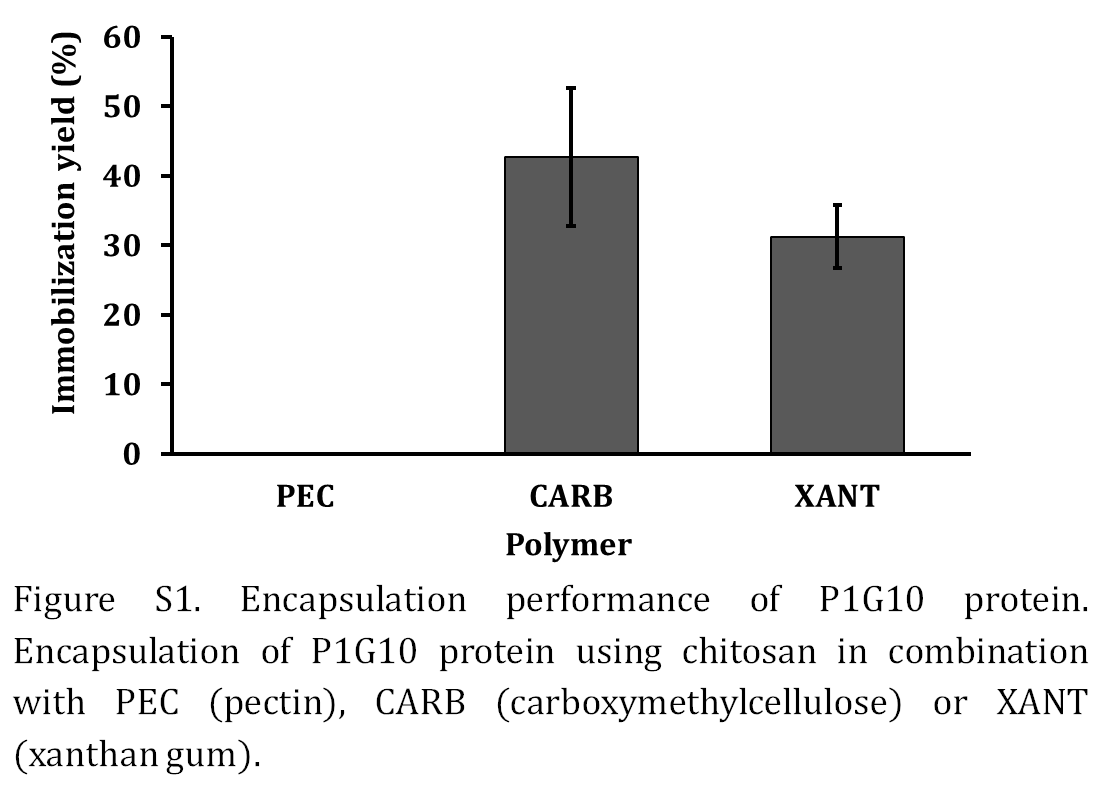

Supplement: Supplementary file 1 [file molecules-30-03747-s001.zip › Figure S1.tif]

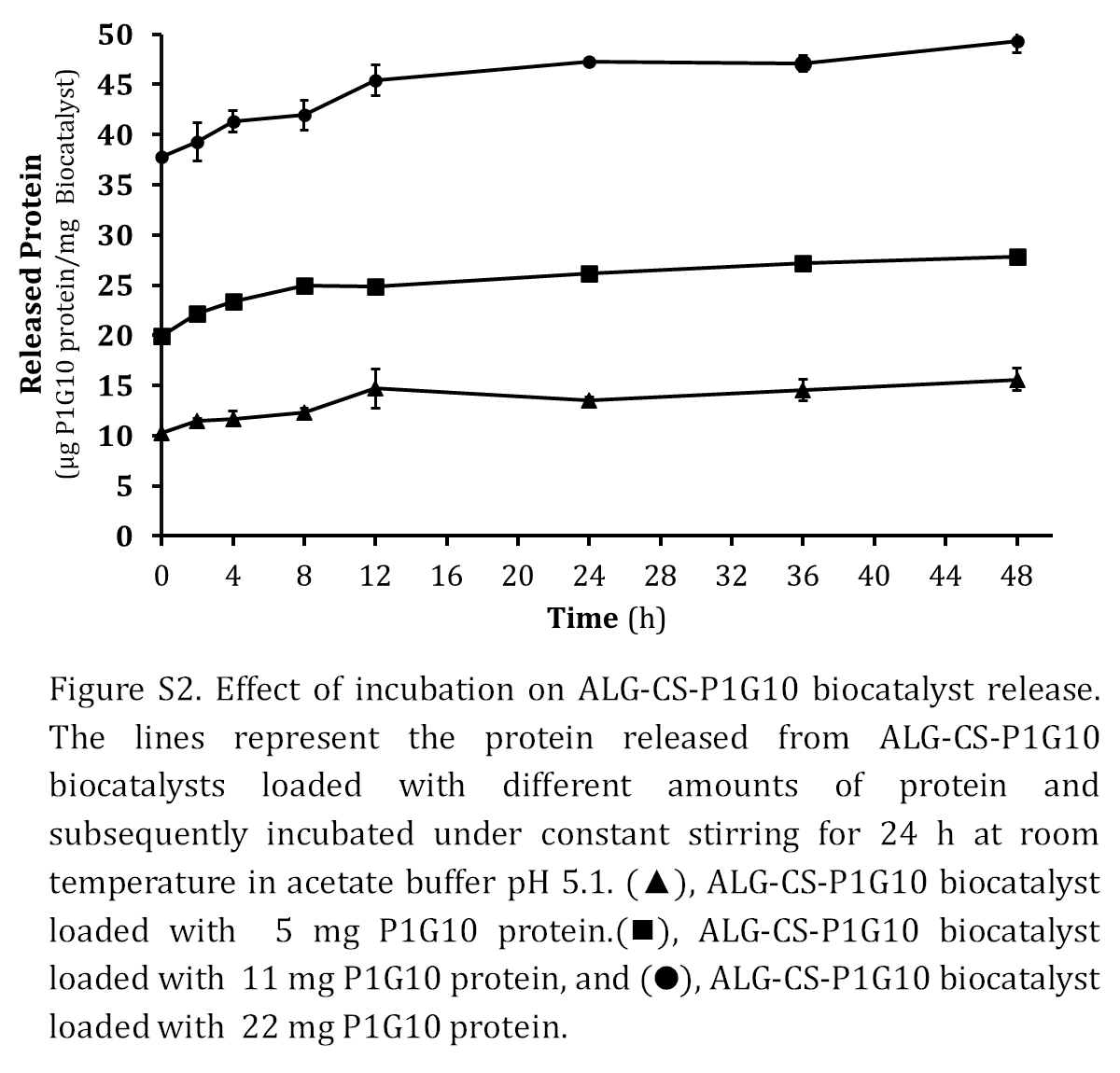

Supplement: Supplementary file 1 [file molecules-30-03747-s001.zip › Figure S2.tif]

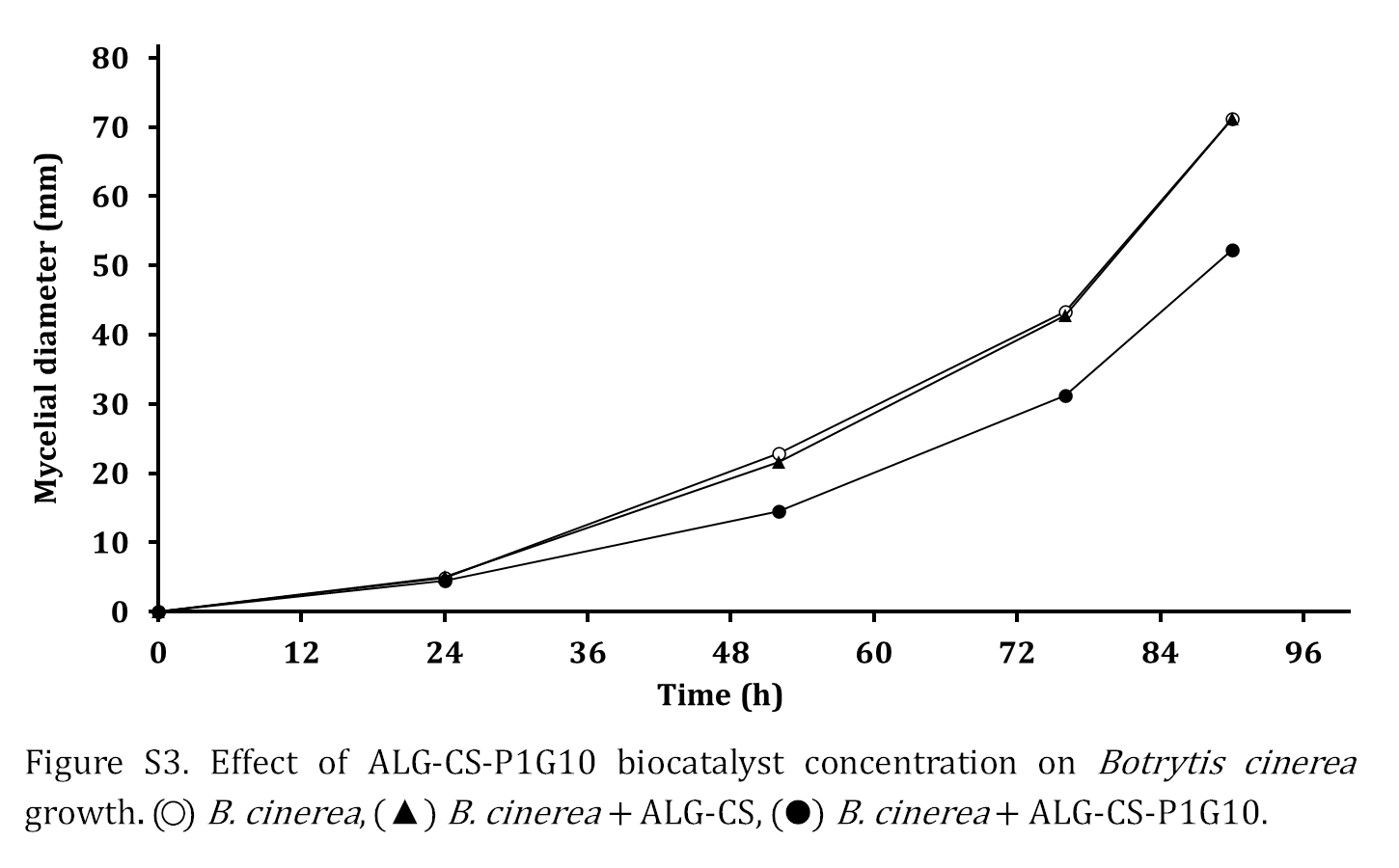

Supplement: Supplementary file 1 [file molecules-30-03747-s001.zip › Figure S3.tif]

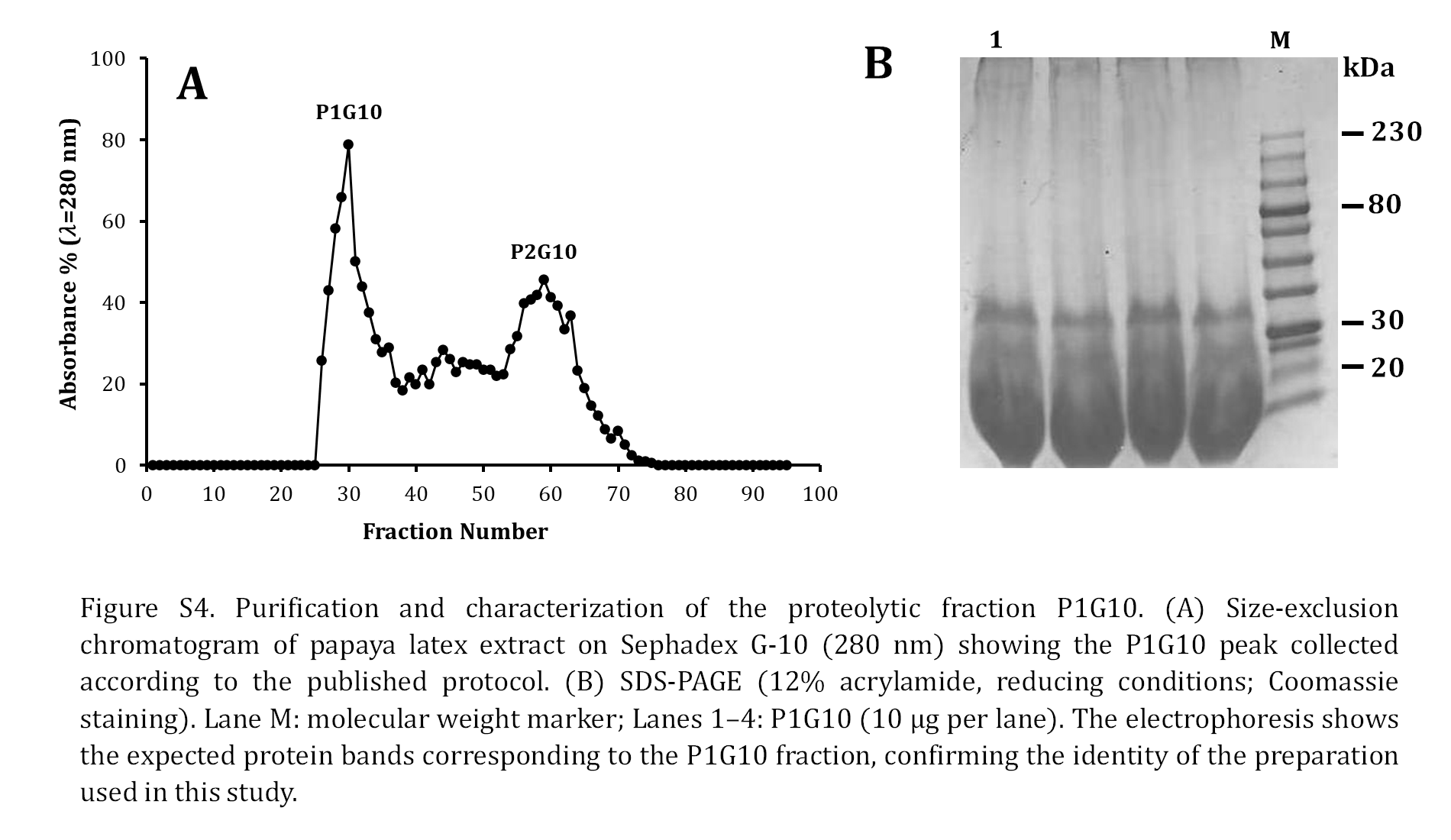

Supplement: Supplementary file 1 [file molecules-30-03747-s001.zip › Figure S4.tif]

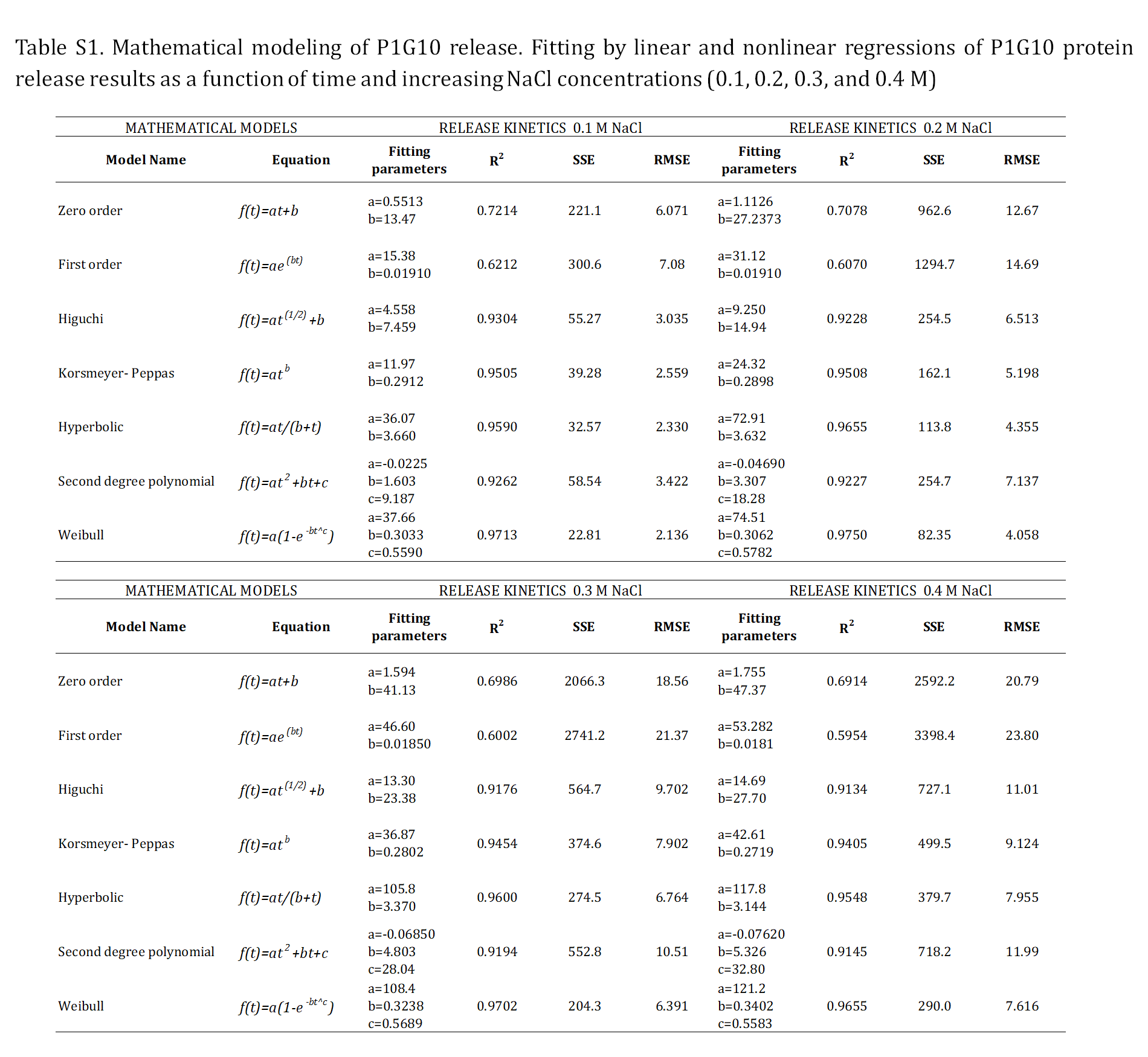

Supplement: Supplementary file 1 [file molecules-30-03747-s001.zip › Table S1.tif]

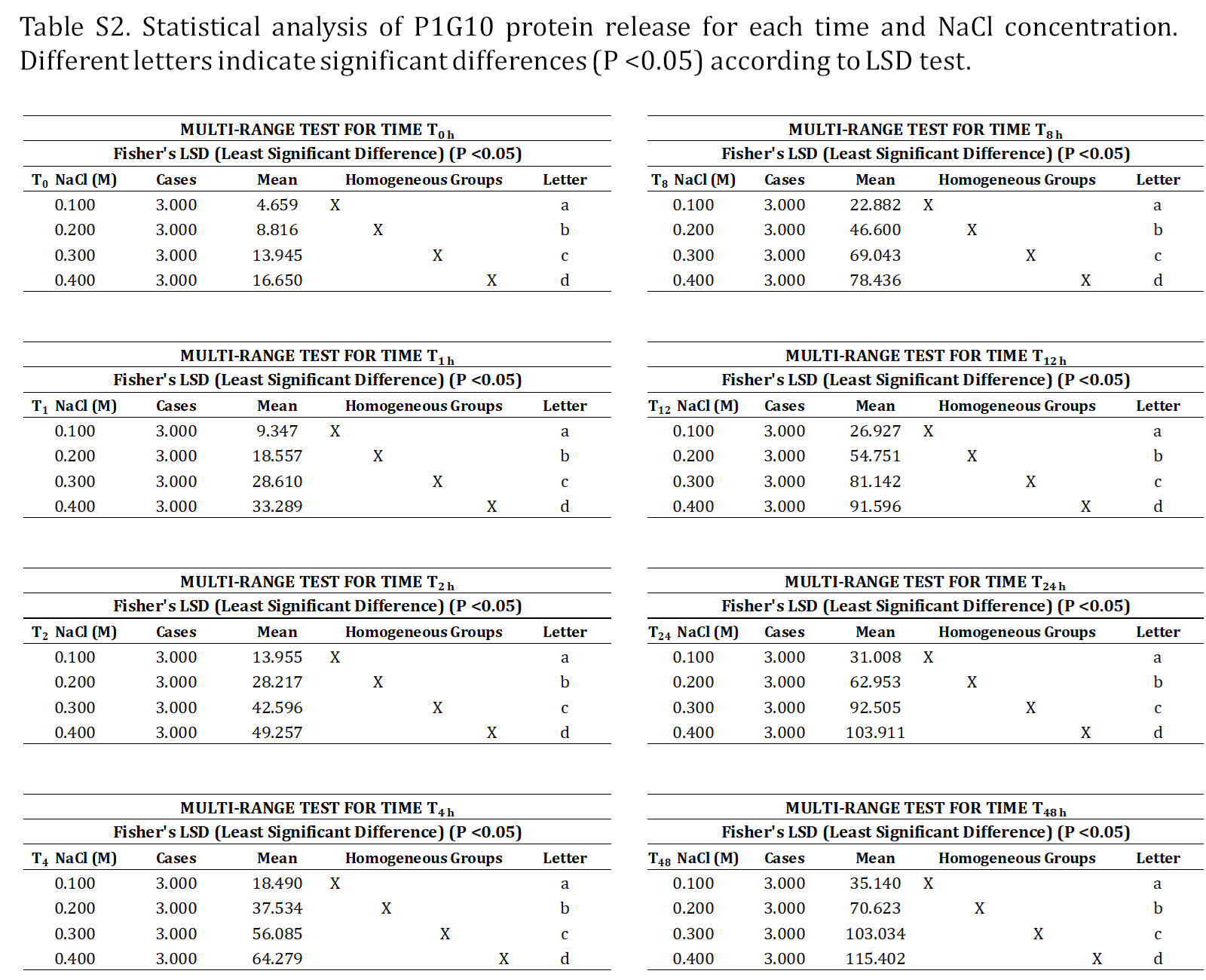

Supplement: Supplementary file 1 [file molecules-30-03747-s001.zip › Table S2.tif]
